# msproteomics sitereport: reporting DIA-MS phosphoproteomics experiments at site level with ease

**DOI:** 10.1093/bioinformatics/btae432

**Published:** 2024-06-29

**Authors:** Thang V Pham, Alex A Henneman, Nam X Truong, Connie R Jimenez

**Affiliations:** Amsterdam UMC location Vrije Universiteit Amsterdam, OncoProteomics Laboratory, Medical Oncology, De Boelelaan 1117, Amsterdam, the Netherlands; Cancer Center Amsterdam, Imaging and Biomarkers, Amsterdam, the Netherlands; Amsterdam UMC location Vrije Universiteit Amsterdam, OncoProteomics Laboratory, Medical Oncology, De Boelelaan 1117, Amsterdam, the Netherlands; Cancer Center Amsterdam, Imaging and Biomarkers, Amsterdam, the Netherlands; Thuyloi University, Faculty of Computer Science and Engineering, 175 Tay Son, Hanoi, Vietnam; Amsterdam UMC location Vrije Universiteit Amsterdam, OncoProteomics Laboratory, Medical Oncology, De Boelelaan 1117, Amsterdam, the Netherlands; Cancer Center Amsterdam, Imaging and Biomarkers, Amsterdam, the Netherlands

## Abstract

**Summary:**

Identification and quantification of phosphorylation sites are essential for biological interpretation of a phosphoproteomics experiment. For data independent acquisition mass spectrometry-based (DIA-MS) phosphoproteomics, extracting a site-level report from the output of current processing software is not straightforward as multiple peptides might contribute to a single site, multiple phosphorylation sites can occur on the same peptides, and protein isoforms complicate site specification. Currently only limited support is available from a commercial software package via a platform-specific solution with a rather simple site quantification method. Here, we present sitereport, a software tool implemented in an extendable Python package called msproteomics to report phosphosites and phosphopeptides from a DIA-MS phosphoproteomics experiment with a proven quantification method called MaxLFQ. We demonstrate the use of sitereport for downstream data analysis at site level, allowing benchmarking different DIA-MS processing software tools.

**Availability and implementation:**

sitereport is available as a command line tool in the Python package msproteomics, released under the Apache License 2.0 and available from the Python Package Index (PyPI) at https://pypi.org/project/msproteomics and GitHub at https://github.com/tvpham/msproteomics.

## 1 Introduction

Protein phosphorylation is one of the most important regulatory mechanism of cellular signaling in biological systems. Profiling phosphorylation states of biological samples is feasible by mass spectrometry-based proteomics. A recent approach called data independent acquisition (DIA-MS) offers advantages for site localization (Bekker-Jensen *et al.* 2020) and data completeness in comparison to the traditional data dependent acquisition (DDA-MS). However, current major DIA-MS data processing software tools, namely Spectronaut (Biognosys) and DIA-NN (Demichev *et al.* 2020), do not offer a common reporting scheme for phosphosites and phosphopeptides, which makes it difficult for downstream analysis and accessing the performance of the processing pipelines. Furthermore, the source code for data reporting is not available for inspection when a large discrepancy is observed (Skowronek *et al.* 2022).

In their seminal work on DIA-MS phosphoproteomics, Bekker-Jensen *et al.* provide a software plugin for Perseus (Tyanova *et al.* 2016), which creates site and peptide level report similar to that of MaxQuant (Cox and Mann 2008) for DDA data. The software was adopted in the Spectronaut software (Biognosys) for site-level reporting. However, the plugin does not support later versions of Spectronaut or other software such as DIA-NN; and as a result, the user needs to resort to in-house scripts (Kitata *et al.* 2021, Skowronek *et al.* 2022). Furthermore, site quantification is currently done by summing up associated intensities, ignoring missing values and not taking into account differences such as ionization efficiency of fragmentation of observed peptides. Here, we present an open-source software tool called sitereport for both site-level and peptide-level reporting for DIA-MS phosphoproteomics. In particular, we implement the MaxLFQ algorithm (Cox *et al.* 2014) for quantification as in the R package *iq* (Pham *et al.* 2020), allowing quantification using peptide intensity (MS1), or peptide fragment intensity (MS2), or both sources of information, which has demonstrated excellent performance for protein expression for DIA-MS experiments.

## 2 Implementation

The input of the software is a long-format report as exported by DIA-MS processing software Spectronaut and DIA-NN. We support MS/MS fragment level exports to allow quantification using MS2 information. We implement data format conversion to accommodate different processing tools. A Spectronaut export can be processed without alteration, while for DIA-NN we provide a tool to adapt the output to a required format, taking into account the input proteome used either in the library-free search or in the library construction phase to properly map detected peptides to protein isoforms.

### 2.1 Phosphosite report

**Figure 1.**
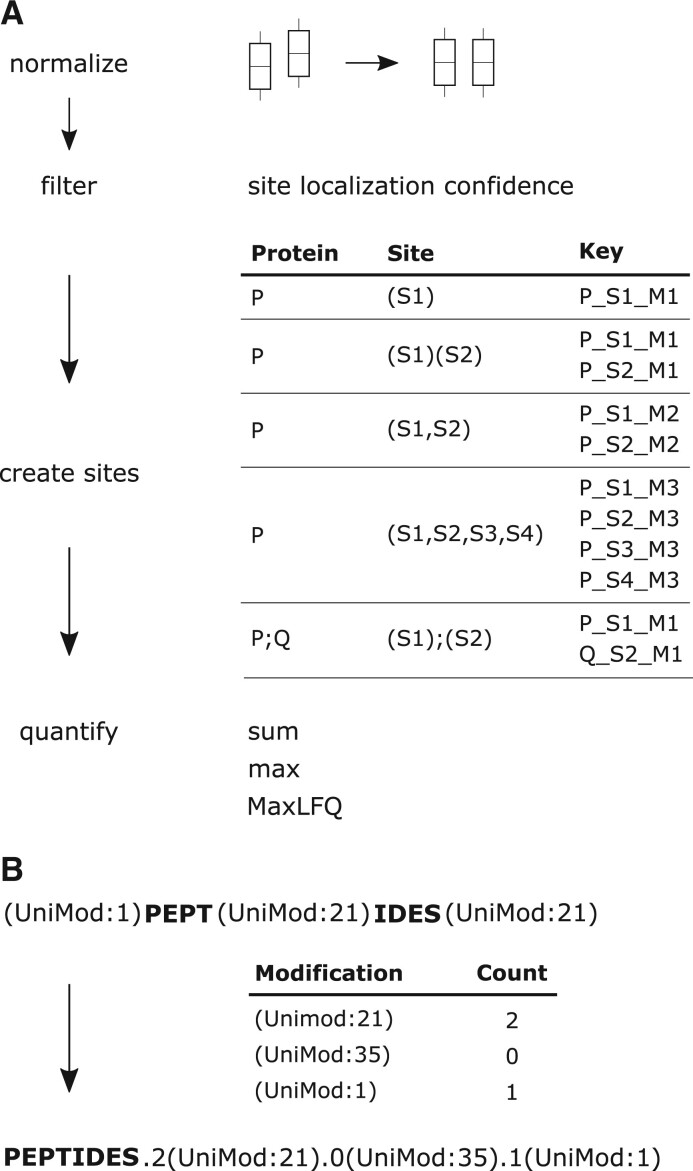
Processing steps of sitereport. (A) Site-level report. The first normalization step is optional. The filtering step is to obtain the so-called class I phosphopeptides with well localized sites. The next step is to form phosphosite keys according to different scenarios (see text for detail). Finally, each phosphosite is quantified by a user-selected method. (B) Peptide-level report. The numbers of specific modifications are appended to the unmodified version of the peptide.

Figure 1A outlines four main steps in sitereport. The first step is optional that normalizes the input data by bringing the median intensities equal across all samples. This is typically used to adjust for variation in the mount of input materials.

In the second step, we filter out peptides with low confidence in identification and localization, e.g. peptide q-value <0.01 and site localization at least 0.75. The filtering on site localization confidence might differ among software tools. Lou *et al.* suggest that a threshold of 0.75 on site localization in Spectronaut is equivalent to a threshold of 0.01 in DIA-NN.

In the third step, we construct phosphosite identifiers. Each identifier is composed of a protein identifier, the site position in the protein, and the so-called phosphorylation multiplicity. The concept of phosphorylation multiplicity originates from MaxQuant in which 1 denotes single phosphorylation, 2 for double phosphorylation of a peptide, and 3 for three or more phosphorylation sites. We consider five scenarios (i) a singly phosphorylated peptide mapped to a single protein, (ii) phosphorylated peptides mapped to multiple locations of a single protein, (iii) a doubly phosphorylated peptides, (iv) peptides phosphorylated at three or more locations, and (v) peptides mapped to multiple protein isoforms. Following this strategy for phosphosite identifier construction, we obtain identical result as the pivot report (wide format) from the latest Spectronaut release (version 18).

Finally, for each site identifier, we form a quantitative table of associated, observed intensities, and subsequently perform site-level quantification. The options are summing the intensities, taking the maximal intensities, and MaxLFQ quantification. Here we have integrated the fast implementation of the MaxLFQ algorithm from R package *iq* (Pham *et al.* 2020) for protein summarization.

### 2.2 Phosphopeptide report

The steps for peptide-level reporting are similar to those of site level reporting except that site localization confidence is optional and another strategy for phosphopeptide identifiers is used. We follow the scheme described by Bekker-Jensen *et al.* (2020) to produce modification-specific peptides. Specifically, for each modified peptides, the number of sites for each modification are appended to the unmodified version as illustrated in Fig. 1B. After the creation of modified peptide keys, we populate the observation for each key and perform quantification like for site quantification.

## 3 Example usage

### 3.1 Phosphosite and phosphopeptide reporting

To demonstrate the usage of the sitereport, we analyze a dataset from the study in (Skowronek *et al.* 2022). Here the authors observed a considerable difference between Spectronaut and DIA-NN. First, we process the outputs from Spectronaut 16 and DIA-NN 1.8 as published in dataset PXD034128 in the ProteomeXchange repository. Spectronaut output can be used immediately. For DIA-NN, we convert the output to another text format where fragment intensities are unrolled into multiple rows. In addition, the proteome used in the library construction stage is used to properly locate phosphosite positions in the proteins. The output can be processed by activating a single command

sitereport report.tsv -tool sn

**Figure 2.**
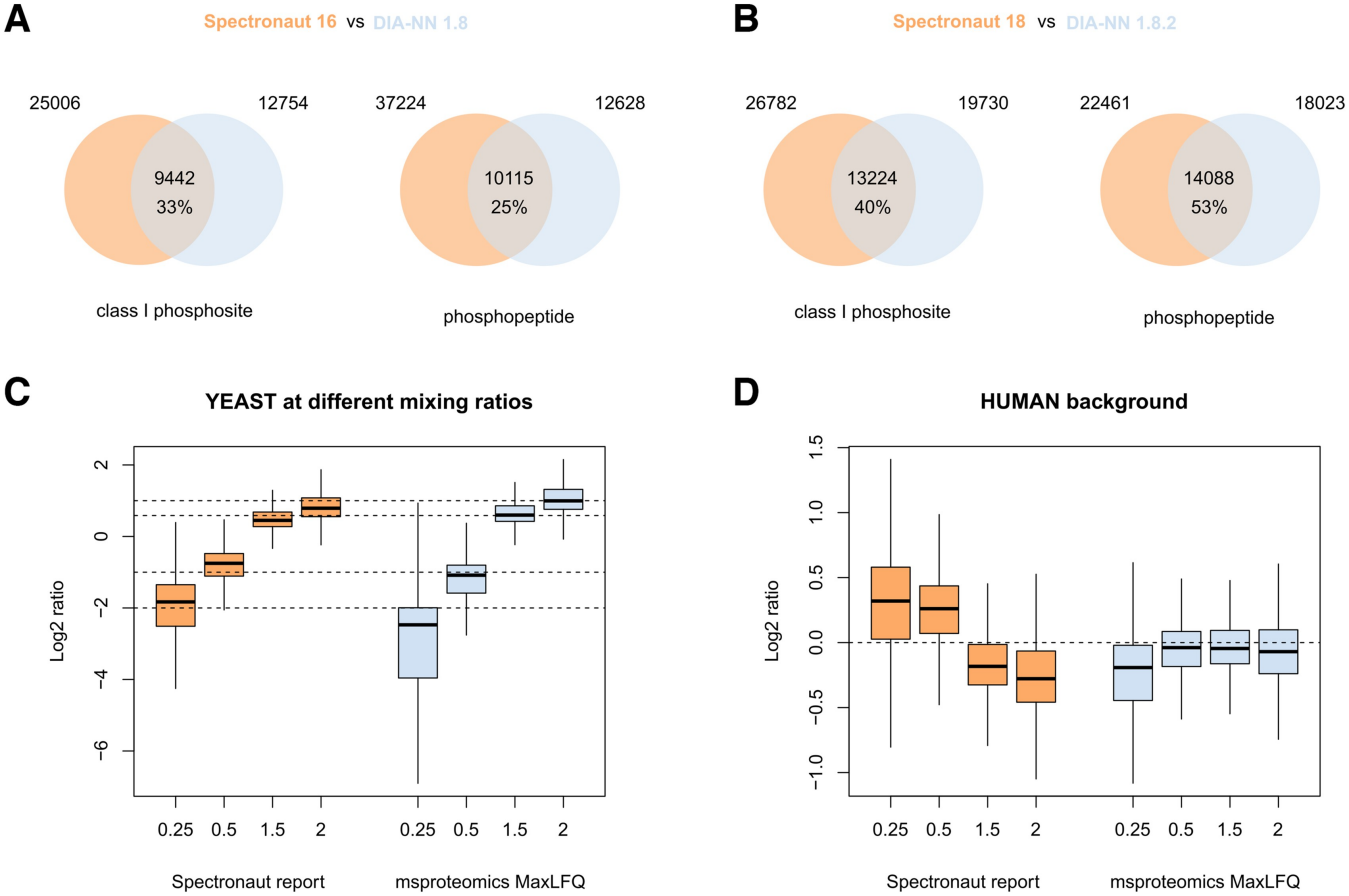
(A) Results of sitereport processing of outputs of Spectronaut 16 and DIA-NN 1.8 of a set of four phosphoproteomics measurements of EGF-stimulated HeLa cells. (B) The results of sitereport of the same set of samples using newer versions of Spectronaut 18 and DIA-NN 1.8.2 beta 27. (C) Quantification results using sitereport of a phosphoproteomics experiment of yeast samples spiked in a human background at different concentrations. The boxplots are the distribution of ratios of phosphosite abundances between conditions. The dashed horizontal lines are the log2 of the expected ratios 0.25, 0.5, 1.5, and 2. (D) Quantification results of the phosphosites in the human background, where the dashed horizontal line indicates the expected equal abundances.

Figure 2A shows the results of sitereport processing. The number of phosphosites are similar to those reported by the authors of the datasets, confirming that Spectronaut results in more phosphosites than DIA-NN. Note that for DIA-NN, we used a threshold of 0.01 for site localization confidence cut-off as suggested in (Lou *et al.* 2023). Nevertheless, we obtain a lower number of phosphosites for Spectronaut (25 006 versus 28 980) and more phosphosites for DIA-NN (12 754 versus 10 510) with more overlapped sites (33% versus 26%).

To verify our implementation, we examine the site-level report produced by Spectronaut 18 (the pivot report). We obtain an identical list of phosphosites for this dataset and all other datasets we have considered. Next, we ran the dataset through the latest peptide collapse plugin (Perseus 2.0.11.0, plugin 1.4.4) with an identical setting used by sitereport. Specifically, we used column EG.PTMAssayProbability for site localization confidence, PG.ProteinGroups for protein identifiers, and with all modifications [Phospho (STY)], [Acetyl (Protein N-term)], [Carbamidomethyl (C)], and [Oxidation (M)]. We have discovered that the Perseus plugin misses several phosphosites due to repetition of peptides in the protein sequence (scenario 2) and multiple isoforms (scenario 5). The data and analysis scripts are publicly available for inspection.

Next, we assess the performance of the latest releases of the processing software. For Spectronaut 18, we process the DIA data with a spectral library created from the DDA data. For the latest DIA-NN 1.8.2 beta 27, we use the library construction pipeline FragPipe platform version 20.0 (https://fragpipe.nesvilab.org/) (Demichev *et al.* 2022, Yu *et al.* 2023) with MSFragger 3.8 (Kong *et al.* 2017), MSBooster 1.1.11 (Yang *et al.* 2023), Percolator 3.06.0 (Käll *et al.* 2007), and the spectral library building module EasyPQP 0.1.40 (https://github.com/grosenberger/easypqp). Figure 2B shows a significant increase in the number of phosphosites and phosphopeptides for DIA-NN, from 12 754 sites to 19 730 sites and 12 628 peptides to 18 023 peptides. There is a small increase in the number of identified phosphosites for Spectronaut, but a large decrease in the number of phosphopeptides. Overall, while Spectronaut 18 still gives a higher number of phosphosites and phosphopeptides than DIA-NN 1.8.2 beta 27, the difference is not as pronounced as that between the previous versions. Note that the true false discovery rates of Spectronaut and the different versions of DIA-NN are unknown since there is no unbiased external control available. Therefore, the differences reported here might be due to different approaches to false discovery rate control.

### 3.2 Site quantification

The package supports different methods for quantification. To demonstrate this feature, we process dataset PXD014525 published in (Bekker-Jensen *et al.* 2020) using Spectronaut 18. The dataset consists of 36 DIA-MS phosphoproteomics runs of mixed species in 5 conditions called yeast 25, yeast 50, yeast 100, yeast 150, and yeast 200 with 6 replicates each (the remaining 6 runs are yeast only and human only). For each phosphosite, the average values in conditions yeast 25, yeast 50, yeast 150, and yeast 200 are divided by the corresponding values in yeast 100, resulting in expected ratios of 0.25, 0.5, 1.5, and 2.0 for yeast phosphosites, and 1.0 for phosphosites belonging to the human background. We used the directDIA mode in Spectronaut 18 with human proteome from UniProt (downloaded 30 March 2023, 42 420 entries), a yeast proteome from UniProt (downloaded 3 May 2023, 6757 entries), and Biognosys iRT peptide sequences. Figure 2C shows the quantification results for yeast phosphosites by the Spectronaut export and by sitereport with MaxLFQ, and Fig. 2D the results for phosphosites in the human background. It can be seen that relative site quantification using MS2 fragments, giving comparable results as those reported by Spectronaut. For the 0.25 ratio comparison, quantification of yeast phosphosites is better by Spectronaut while quantification of the human background is better by sitereport. For other group comparisons, the results by sitereport are better for both yeast and human background. Supplementary Figure S1 shows the quantitative results stratified by phosphorylation multiplicity. Supplementary Figures S2 and S3 show the results of the three quantification methods with and without normalization. Here, the normalization step appears to have a large effect in quantification for phosphosites in the human background.

## 4 Conclusion

We have implemented a reference reporting scheme for DIA-MS phosphoproteomics experiments for both site-level and peptide-level quantification. The Python software package currently supports two major DIA-MS processing software tools Spectronaut and DIA-NN, and readily extendable to support other tools via format conversion. Three quantification methods, summing associated intensities, taking the maximal value, and MaxLFQ, are implemented together with data normalization. Our software will facilitate downstream data analysis at the phosphosite level and aid in benchmarking upstream data processing tools.

## Supplementary Material

btae432_Supplementary_Data

## Data Availability

The data used in this article are publicly available at doi.org/10.5281/zenodo.11494771.
