# Supplementary material for: msproteomics sitereport: reporting DIA-MS phosphoproteomics experiments at site level with ease
Source: Bioinformatics. 2024 Jun 29;40(7):btae432. doi: 10.1093/bioinformatics/btae432 (PMC11239223; doi:10.1093/bioinformatics/btae432)
Supplement: btae432_Supplementary_Data [file btae432_supplementary_data.pdf]

SUPPLEMENTARY INFORMATION

msproteomics sitereport: reporting DIA-MS phosphoproteomics experiments at site level with ease

Thang V Pham[1], Alex A Henneman[1], Nam X Truong[2], Connie R Jimenez[1]

[1]Amsterdam UMC, Vrije Universiteit Amsterdam, OncoProteomics Laboratory, Medical Oncology, De Boelelaan 1117, 1081 HV, The Netherlands

[2]Thuyloi University, Faculty of Computer Science and Engineering, 175 Tay Son, Hanoi, Vietnam

Phosphorylation multiplicity

Extracting a site-level report is not straightforward as multiple phosphopeptides might contribute to a single site and multiple sites can be phosphorylated on a single peptide. We follow a strategy in (Bekker-Jensen et al., 2020) that implements a scheme in MaxQuant (Cox & Mann, 2008) to handle phosphorylation multiplicity. It considers three categories, namely phosphopeptides with a single phosphorylated site, phosphopeptides with two phosphorylated sites, and phosphopeptides with more than two phosphorylated sites.

For example, consider two overlapping phosphopeptides called `PEP`**T**(phospho)`ITSK` and `PEPKPEP`**T**(phospho)`ITSK` detected, both pointing to the same amino acid **T** at position 4 on the first peptide and position 8 on the second peptide. A unique entry will be created for this phosphosite, using the position of the specific protein isoform that both peptides belong to. Furthermore, this site has multiplicity of 1. All quantities belonging to the two phosphopeptides will be populated for quantification.

Next consider a doubly phosphorylated peptide `PEP`**T**(phospho)`I`**T**(phospho)`SK`. This phosphopeptide will result in two entries according to scenario 3 in the main text Fig. 1. The same peptide when triply phosphorylated `PEP`**T**(phospho)`I`**T**(phospho)**S**(phospho)`K` will result in three other entries according to scenario 4. In total, 5 different phosphosite entries will be created. Hence, a single phosphosite might be present in multiple rows in the site report. Peptide fragment intensities related to each entry will be used for quantification, and hence the same quantitative information might be used multiple times.

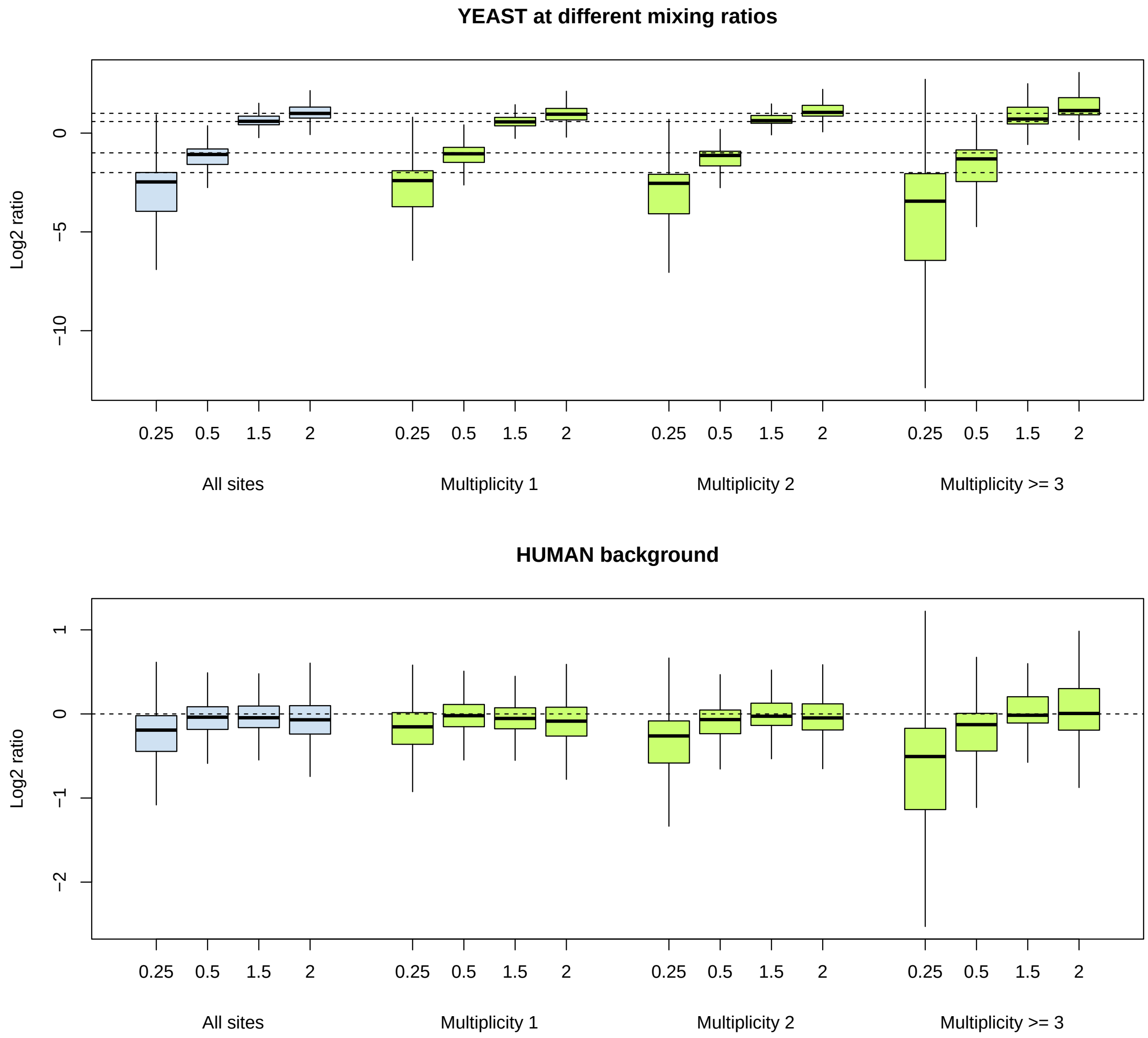

Supp. Fig. 1: Quantification results of yeast phosphosites spiked in a human background at different concentrations, stratified by phosphorylation multiplicity. The boxplots are distributions of ratios of phosphosite abundances between conditions. In the top panel, the dashed horizontal lines are the log2 of the expected ratios 0.25, 0.5, 1.5, and 2. The bottom panel displays the results of the phosphosites in the human background with an expected log2 ratio of 0 for all comparisons.

Supp. Fig. 1 shows the result of the Bekker-Jensen dataset stratified by different multiplicity categories. It can be seen that all categories exhibit expected regulation for the yeast fraction and the human background, and the multiplicity 3 group showing somewhat more variation than the other two.

Supp. Tab. 1: Example of three methods for quantification: 'sum', 'max', and MaxLFQ

	**Sample 1**	**Sample 2**	**Sample 3**
**Intensity 1**	16	4	missing
**Intensity 2**	4	missing	16
Quantification result			
method 'sum'	20	4	16
method 'max'	16	4	16
method MaxLFQ	8	2	32

Site quantification

For site quantification, we populate all fragment intensities associated with a phosphosite in a tabular format. We implement three quantification methods: the 'sum' method adding up all intensities, the 'max' method taking the maximal intensity, and the MaxLFQ method (Cox & Mann, 2008; Pham et al., 2020). Supp. Tab. 1 presents an example to illustrate the different methods. Suppose that two phosphopeptides for a particular site are detected across three samples. The first peptide is detected only in sample 1 and sample 2 with intensity of 16 and 4 (intensity unit), respectively. The second peptide is detected only in sample 1 and sample 3 with intensity of 4 and 16, respectively.

The 'sum' method will add up all detected intensities for each sample, resulting in quantitative values of 20, 4, and 16 for the three samples. The 'max' method results in values of 16, 4, and 16, respectively. For both methods, the intensities of sample 2 and sample 3 were derived from two different peptides, which is not suitable for comparison because of the potential difference in ionization efficiency of the two peptides.

The MaxLFQ method considers pairwise ratios between samples. In this example, according to the first peptide, sample 1 is 4–fold higher than sample 2, and according to the second peptide, the first sample is 4–fold less than sample 3. There is no direct observed ratio between sample 2 and 3. The algorithm will find a set of values best fit these ratios, and in this example, there is a perfect fit of 8, 2, and 32. Following the algorithm in (Pham et al, 2020), the calculation was performed in a log2 space, in which the result was scaled so that the mean of the estimated intensities and the mean of the observed intensities are

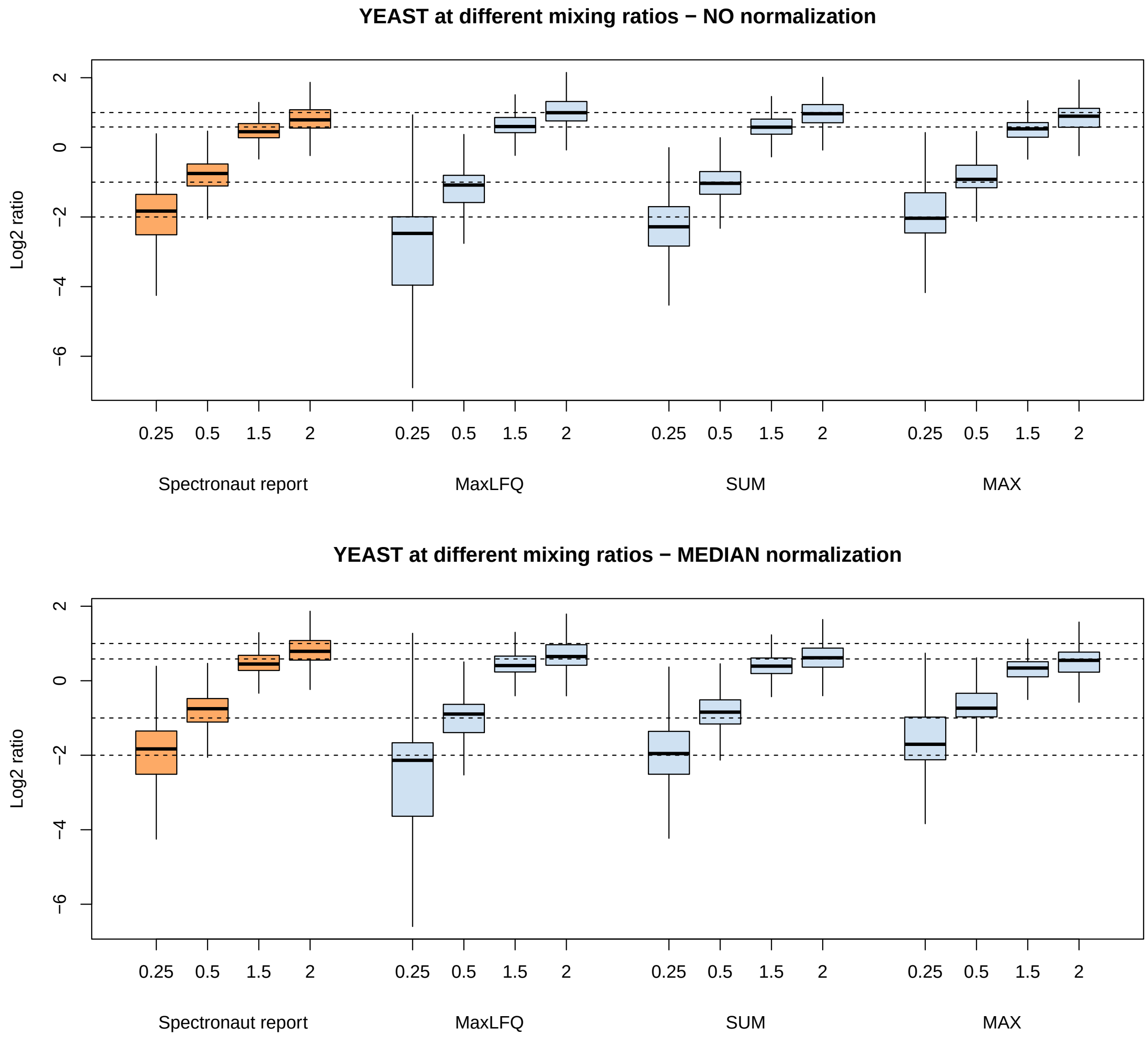

Supp. Fig. 2: Quantification results of yeast phosphosites spiked in at different concentrations by different quantification methods. The boxplots are distributions of ratios of phosphosite abundances between conditions. The dashed horizontal lines are the log2 of the expected ratios 0.25, 0.5, 1.5, and 2. In the top panel, no normalization was performed. In the bottom panel, the samples were aligned by their median intensities.

equal. Specifically, in the log2 space, the input intensities are 4, 2, NA (missing value) in the first row, and 2, NA, 4, in the second row, resulting in an average of 3 per intensity value excluding the NA's. The estimated result in the log2 space is 3, 1, and 5, which also has an average of 3 per sample.

Supp. Fig. 2 and Supp. Fig. 3 show the results of the three quantification methods with and

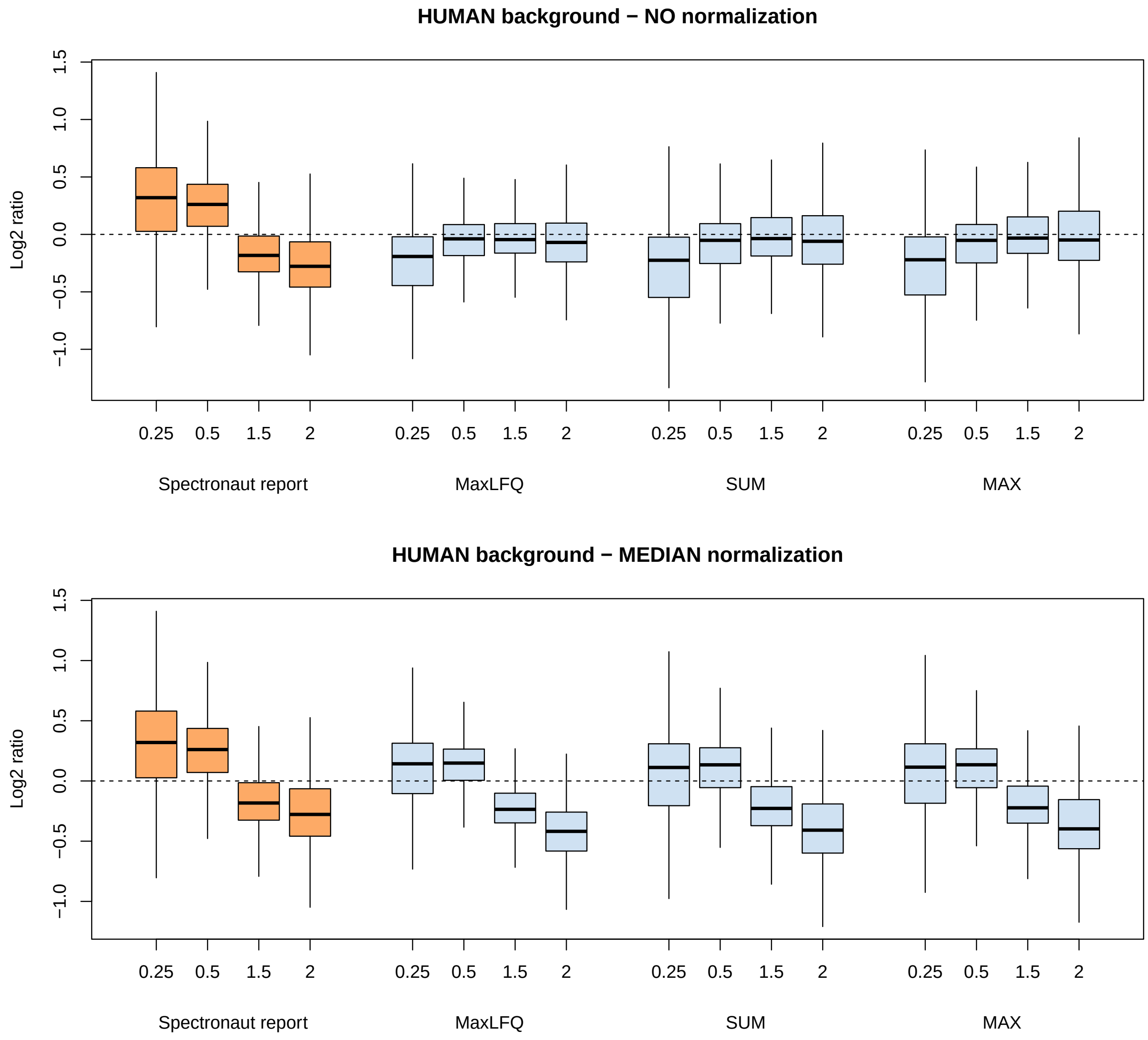

Supp. Fig. 3: Quantification results of the phosphosites in the human background in a spiked-in experiment. The boxplots are distributions of ratios of phosphosite abundances between conditions. The dashed horizontal lines are the log2 of the expected ratio of 0 for the phosphosites in the background. In the top panel, no normalization was performed. In the bottom panel, the samples were aligned by their median intensities.

without normalization on the example dataset (Bekker-Jensen et al., 2020). It can be seen that the three methods have similar performance on this dataset and normalization appears to have a larger effect.

Package installation

The package can be installed from the standard Python package management *pip*. One command line statement is sufficient, that is *pip install msproteomics*. Nevertheless, it is likely that the user should create a separate environment for testing.

```
> conda create -n msproteomics
> conda activate msproteomics
```

On some system, the user might need to first install the Python package *pybind11*.

```
> pip install pybind11
> pip install msproteomics
```

Package usage

Once the package has been successfully installed, the user should be able to see the full set of parameters by the command

```
> sitereport -h
```

The experimental data are available in the public Zenodo repository with the object identifier 10.5281/zenodo.11494771. It contains the dataset in (Bekker-Jensen et al., 2020), re-processed by Spectronaut 18. To create a phosphosite report, we run a command line

```
> sitereport olsen-long-format-ptm-report.tsv -tool sn
```

The software will display the default settings used for Spectronaut. For quantification, two important parameters are the normalization method and quantification method. The former can be set by the switch *-normalize* with value *none* or *median*, and the latter by *-quant_method* with value *sum*, *max*, or *maxlfq*. The underlying data for Supp. Fig. 2 and Supp. Fig. 3 were obtained by the different combinations of normalization methods and quantification methods. For instance, the following command is used for median normalization together with the quantification method 'max'.

```
> sitereport olsen-long-format-ptm-report.tsv -tool sn -normalize median
-quant_method max
```

Data format

A DIA-NN report is first converted to a generic sitereport format. We will use the published dataset PXD034128 (Skowronek et al., 2022) in the ProteomeXchange repository for illustration.

```
> read_diann -o report_msproteomics.tsv -f
uniprot-reviewed_yes_AND_organism__Homo_sapiens__Human___9606___--.fasta
Phospho_EGF_diAID.tsv
```

This will produce an output file *report_ msproteomics.tsv* in a generic format for sitereport. The input file should contain a set of specified column names. If found, these columns are copied to the output. The expected columns are `File.Name`, `Run`, `Protein.Group`, `Protein.Ids`, `Genes`, `Modified.Sequence`, `Stripped.Sequence`, `Precursor.Id`, `Q.Value`, `Global.Q.Value`, `Proteotypic`, `Ms1.Area`, `Fragment.Quant.Corrected`, `PTM.Q.Value` and `PTM.Site.Confidence`.

Two columns are mandatory and their names can be specified from the command line. The first mandatory input column is `Fragment.Quant.Corrected`, which contains all fragment ion intensities and is simply expanded in the output into one intensity value per row. Output columns `Fragment.Rel.Id` and `Fragment.Intensity` contain an arbitrary generated fragment id number (to distinguish fragments belonging to the same modified peptide) and the expanded fragment intensity value. The other mandatory column is the `Modified.Sequence` column, which is the main input and are row-wise processed into an unmodified peptide backbone sequence and a list of modifications localized on this peptide. For example, the following command uses the column `Fragment.Quant.Raw` in place of `Fragment.Quant.Corrected` for quantitation

```
> read_diann -o report_msproteomics.tsv -E Fragment.Quant.Raw
-f uniprot-reviewed_yes_AND_organism__Homo_sapiens__Human___9606___--.fasta
report.tsv
```

The software digests one or more protein sequence databases into an efficient lookup data structure, and subsequently looks up the unmodified peptide, reconstructing all full protein offsets of the input peptide modifications. In the output the columns `Peptide.Backbone`, `Peptide.Offsets`, `Nr.Mods` and `Nr.Phospho` are appended specifying the peptide sequence

Supp. Tab. 2: A snapshot of the columns generated for the generic sitereport format.

**Protein.Sites**	**Proteins.Fasta**	**Peptide.Backbone**
(nA2,pS11,pS13)	O75822	AAAAAAAGDSDSWDADAFSVEDPVRK
(nA2,pS11,pS13)	O75822	AAAAAAAGDSDSWDADAFSVEDPVRK

**Phospho.Site.Specs**	**Fragment.Rel.Id**	**Fragment.Intensity**
(S11,S13)	1	1488.02
(S11,S13)	2	1479.02

that is looked up, the offsets of the modifications, the number of modifications and how many of these are phosphorylations, respectively. If the lookup fails, the row will be excluded from the output. If it succeeds, the columns `Fasta.Files` and `Proteins.Fasta` contain the protein sequence file(s) and protein names in which the peptide backbone is found.

The columns `Protein.Sites` and `Phospho.Site.Specs` contain the offsets of all modifications and only phosphorylations, respectively, for each entry in the `Proteins.Fasta` column. In these columns, for each protein, parentheses enclose a comma-separated list of amino-acids full protein offset specifications. Multiple proteins in `Proteins.Fasta` lead to a semi-colon separated list of such offset lists. In `Protein.Sites`, the type of modification is specified by a small case letter prepended to amino acid offsets. The symbols `n`, `p`, `m`, `d`, `u`, `g`, `t`, `e`, `q` and `a` denote `UniMod:1`, `UniMod:21`, `UniMod:35`, `UniMod:36`, `UniMod:121`, `UniMod:34`, `UniMod:37`, `UniMod:27`, `UniMod:28` and `UniMod:385`, respectively. The symbol `x` is used for any unrecognized modifications. Supp. Tab. 2 displays an example snapshot of the output.

Once the report has been converted to the generic sitereport format, we can obtain the final report by

```
> sitereport report_msproteomics.tsv
```

In short, it is straightforward to support new processing software by developing a suitable format conversion tool.
